# Obesity Due to Steroid Receptor Coactivator-1 Deficiency Is Associated With Endocrine and Metabolic Abnormalities

**DOI:** 10.1210/clinem/dgac067

**Published:** 2022-02-08

**Authors:** Tessa M Cacciottolo, Elana Henning, Julia M Keogh, Pierre Bel Lassen, Katherine Lawler, Rebecca Bounds, Rachel Ahmed, Aliki Perdikari, Edson Mendes de Oliveira, Miriam Smith, Edmund M Godfrey, Elspeth Johnson, Leanne Hodson, Karine Clément, Agatha A van der Klaauw, I Sadaf Farooqi

**Affiliations:** 1 University of Cambridge Metabolic Research Laboratories and NIHR Cambridge Biomedical Research Centre, Wellcome-MRC Institute of Metabolic Science, Box 289, Addenbrooke’s Hospital, Cambridge CB2 0QQ, UK; 2 Sorbonne Université, INSERM, Nutrition and Obesities: Systemic Approaches (NutriOmics) Research Group and Assistance Publique hôpitaux de Paris, Nutrition Department, Pitié-Salpêtrière Hospital, 75013 Paris, France; 3 Department of Radiology, Addenbrooke’s Hospital, Cambridge CB2 0QQ, UK; 4 Oxford Centre for Diabetes, Endocrinology and Metabolism, University of Oxford, Churchill Hospital and National Institute for Health Research Oxford Biomedical Research Centre, Oxford University Hospitals Foundation Trust, Headington, Oxford OX3 7LE, UK

**Keywords:** SRC-1, nuclear hormone receptors, obesity, hormone resistance

## Abstract

**Context:**

Genetic variants affecting the nuclear hormone receptor coactivator steroid receptor coactivator, *SRC-1,* have been identified in people with severe obesity and impair melanocortin signaling in cells and mice. As a result, obese patients with *SRC-1* deficiency are being treated with a melanocortin 4 receptor agonist in clinical trials.

**Objective:**

Here, our aim was to comprehensively describe and characterize the clinical phenotype of *SRC-1* variant carriers to facilitate diagnosis and clinical management.

**Methods:**

In genetic studies of 2462 people with severe obesity, we identified 23 rare heterozygous variants in *SRC-1*. We studied 29 adults and 18 children who were *SRC-1* variant carriers and performed measurements of metabolic and endocrine function, liver imaging, and adipose tissue biopsies. Findings in adult *SRC-1* variant carriers were compared to 30 age- and body mass index (BMI)-matched controls.

**Results:**

The clinical spectrum of *SRC-1* variant carriers included increased food intake in children, normal basal metabolic rate, multiple fractures with minimal trauma (40%), persistent diarrhea, partial thyroid hormone resistance, and menorrhagia. Compared to age-, sex-, and BMI-matched controls, adult *SRC-1* variant carriers had more severe adipose tissue fibrosis (46.2% vs 7.1% respectively, *P* = .03) and a suggestion of increased liver fibrosis (5/13 cases vs 2/13 in controls, odds ratio = 3.4), although this was not statistically significant.

**Conclusion:**

*SRC-1* variant carriers exhibit hyperphagia in childhood, severe obesity, and clinical features of partial hormone resistance. The presence of adipose tissue fibrosis and hepatic fibrosis in young patients suggests that close monitoring for the early development of obesity-associated metabolic complications is warranted.

Single-gene disorders that disrupt the development and/or function of the hypothalamic leptin-melanocortin pathway cause hyperphagia (increased food intake), neuroendocrine abnormalities, impaired sympathetic tone, and weight gain from early childhood (1). Establishing a genetic diagnosis is helpful for patients and families, and clinical guidelines recommend genetic testing as part of the diagnostic evaluation of people with severe obesity that begins in childhood (2). Genetic findings increasingly affect clinical care as a number of therapies are now licensed in the United States and Europe for the chronic weight management of people with genetic obesity syndromes (3, 4).

In a previous exome-sequencing study of 2548 European ancestry patients with severe, early-onset obesity (mean body mass index [BMI] SD score > 3; age of onset < 10 years) (5), we reported rare heterozygous variants in the gene-encoding steroid receptor coactivator-1 (SRC-1) (6). SRC-1 is a widely expressed coactivator that modulates the activity of nuclear hormone receptors (NHRs) and other transcription factors; targeted deletion of *SRC-1* causes obesity in mice (7). We showed that in the hypothalamus, SRC-1 interacts with phosphorylated STAT3 (signal transducer and activator of transcription-3) to potentiate leptin-mediated transcription of proopiomelanocortin (POMC) (6); POMC-derived melanocortin peptides signal through the melanocortin-4 receptor (MC4R) to reduce food intake (Fig. 1A). In mice, deletion of *SRC-1* in Pomc neurons decreased *Pomc* expression and increased food intake, leading to obesity (6). Human obesity-associated variants in *SRC-1* impaired leptin-mediated Pomc reporter activity in cells, predominantly through a dominant negative effect. We established a causal link between rare human variants and obesity by characterizing a knockin mouse model of a human loss-of-function (LOF) *SRC-1* variant that exhibited increased food intake and weight gain (6). As a result of these studies, patients with severe obesity and rare *SRC-1* variants are now being recruited into phase 2 clinical trials of setmelanotide, an MC4R agonist, licensed for the chronic weight management of POMC and leptin receptor deficiency (3, 8, 9). As patients with SRC-1 deficiency are increasingly being identified by genetic screening in obesity clinics, and as their identification has potential implications for therapy, the aim of this study is to describe the clinical spectrum seen in patients with rare variants in *SRC-1* to facilitate diagnosis and clinical management.

**Figure 1.**
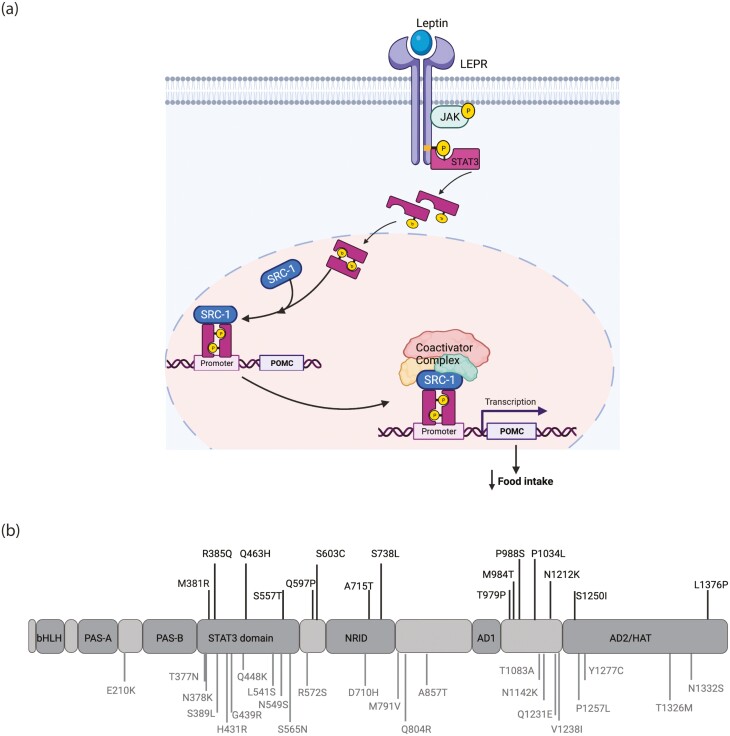
Obesity-associated *SRC-1* variants. A, SRC-1 modulates the leptin signaling pathway. On leptin binding, the leptin receptor (LEPR) phosphorylates JAK2, which in turn activates STAT3. Phosphorylated STAT3 (pSTAT3) dimerizes and translocates to the nucleus, where it binds to the *POMC* promoter. SRC-1 and other coactivators are recruited as part of a coactivator/corepressor complex and initiate transcription of the neuropeptide Pro-opiomelanocortin (POMC), which reduces food intake in the fed state. B, Rare variants in *SRC-1* identified in individuals with severe early-onset obesity. Variants previously reported (6) and found to cause a loss of function are denoted in black and located above the protein; newly identified variants are denoted in gray, below the protein. AD, activating domains 1 and 2; bHLH, basic helix-loop-helix; HAT, histone acetyl transferase domain; NRID, nuclear receptor interacting domain; PAS, Per-Arnt-Sim (A and B) domains; SRC-1, steroid receptor coactivator-1.

In this study, we identified additional carriers of rare variants (global minor allele frequency < 0.1% across all populations) in *SRC-1* using exome sequencing and targeted resequencing of patients with severe obesity (methods reported previously) (5, 10). As well as developing obesity, *SRC-1* knockout mice exhibit resistance to thyroid hormone (RTH) (11) and partial resistance to estrogen, progesterone, and testosterone (12), manifesting as decreased growth and development of the uterus, ovaries, mammary glands, and testes (12) and loss of trabecular bone mass (13). Furthermore, SRC-1 interacts with farnesoid-X receptor (FXR) and liver X receptor (LXR), regulators of de novo lipogenesis (DNL) (14) and with PGC-1α to mediate the activity of PPAR-γ, a master regulator of adipogenesis and thermogenesis in mice (7). Here we explored clinical features in a cohort of 47 *SRC-1* variant carriers who consented to clinical studies, focusing on the metabolic and endocrine phenotypes that may arise from impaired NHR–mediated gene transcription.

## Materials and Methods

### Patients and Study Design

We studied patients with severe obesity (BMI, weight in kilograms divided by height in meters squared) SD score > 3) of early-onset (age < 10 years) recruited to the Genetics of Obesity Study cohort (www.goos.org.uk). These studies were approved by the Cambridge Local Research Ethics Committee (03/103, 03/104, 18/EE/0032) and conducted in accordance with the principles of the Declaration of Helsinki. Each participant or their legal guardian (for children age < 16 years) provided written, informed consent, and minors provided verbal or written consent. Exome sequencing and targeted resequencing were performed as previously described (5); all variants were verified by Sanger sequencing.

We performed a series of studies to describe the clinical features of *SRC-1* variant carriers. We compared data from adult variant carriers with those obtained on obese volunteers recruited by advertisement who were carefully matched for age, sex, and BMI and in whom variants in *SRC-1* were excluded by Sanger sequencing. Those with comorbidities (type 2 diabetes, autoimmune hypothyroidism and hyperthyroidism and/or taking levothyroxine treatment) and concomitant medications that affect body weight and/or metabolism were excluded from clinical studies. Some measurements were not performed on all eligible cases and controls for a variety of reasons (weight limit for dual x-ray absorptiometry [DEXA] and/or magnetic resonance imaging [MRI], challenging vascular access, medications); numbers studied are indicated for each measurement. Eighteen children with variants in *SRC-1* were compared to 11 age-matched controls. Whereas adult controls were matched for BMI, pediatric controls were not, as equally severely obese children often have other genetic conditions.

Adipose tissue samples (Paris obesity cohort) were obtained from people with severe obesity prospectively recruited to the Prospective Bariatric Surgery Cohort of the Nutrition Department at Pitié-Salpêtrière Hospital, Paris, France, between 2014 and 2018. They are part of several studies registered at ClinicalTrials.gov (P050318, NCT01655017, and NCT01454232). Informed consent was obtained to perform paired omental and subcutaneous surgical adipose tissue biopsies obtained during bariatric surgery. In the surgical department in Paris, patients are asked to have a stabilized weight before bariatric surgery and to not routinely consume a restrictive diet preoperatively in contrast to clinical practice at other centers. The details of adipose tissue handling have been reported previously (15).

### Anthropometry, Body Composition, and Liver Imaging

Weight and height were measured barefoot in light clothing. DEXA (DPX software; Lunar Corp) was used to determine body composition, bone mineral content and density (BMD) (whole body). Subcutaneous and visceral fat mass were quantified in a subset of cases and controls by MRI at the level of L1 and analyzed using OsiriX Lite software (Pixmeo). In a subset of 13 *SRC-1* variant carriers and 15 controls, MRI scans were carried out on a wide-bore 1.5-T whole-body system (GE Medical Systems) and included localizer images, T2-weighted fat-saturated images, proton density fat fraction (PDFF), and magnetic resonance elastography (MRE). Participants fasted for 4 hours before the procedure and were positioned in the supine position with an acoustic driver positioned superficial to the liver. Liver volume: T2-weighted images were used to draw regions of interest around the liver perimeter on consecutive slices and volume computed using OsiriX Lite (Pixmeo). Images for PDFF were acquired using a gradient echo sequence with a low flip angle that were then analyzed using the validated IDEAL-IQ software (GE Healthcare) to generate an unbiased quantitative measure of percentage fat fraction. MRE was used to quantify hepatic fibrosis (> 2.9 kPa correlates with statistically significant hepatic fibrosis on histology and a METAVIR score of ≥ F2) (16). MRE images were acquired using a phase-contrast gradient echo sequence. Where participants were unable to have MRI (weight ≥ 230 kg, abdominal girth ≥ 70 cm), acoustic radiation force impulse (ARFI) elastography or mechanical impulse elastography (FibroScan, Echosens) was performed. ARFI was performed using Canon shear wave elastography by a trained radiologist on an Aplio 500 (version 2 software) or Aplio i800. Mechanical impulse elastography was performed with FibroScan using the XL probe. Results were accepted if they met recognized reliability criteria (interquartile range/median < 0.3) (17). Operators were blinded to genotype. Two participants were unable to have an MRI scan (weight ≥ 230 kg, abdominal girth ≥ 70 cm); as such ARFI or transient elastography was performed.

### Metabolic Measurements and Autonomic Function

Ad libitum energy intake was assessed using an 18-MJ meal of known macronutrient content (50% carbohydrate, 30% fat, 20% protein) administered after an overnight fast; energy intake was expressed per kilogram of lean body mass as measured by DEXA to allow comparison between individuals of different body weights and compositions. Basal metabolic rate and respiratory quotient were determined by indirect calorimetry after a 10-hour overnight fast using an open circuit, ventilated, canopy measurement system (Europa Gas Exchange Monitor; NutrEn Technology). After adjustment for body composition, basal metabolic rate was compared to predicted metabolic rate based on standard age- and sex-specific equations. Blood pressure was measured in the rested, fasted state using automated wrist (OMRON Healthcare) monitors. We used a DINAMAP automated brachial monitor with the Dura large adult cuff (GE Healthcare) in 3 people whose wrist circumference precluded use of this device. Heart rate was measured using a digital portable heart rate monitor (Actiheart, Cambridge Technologies). Heart rate data collected using the portable heart rate monitor were exported to MS Excel 2010 via Actiheart software (version 4.0.116, CamNtech Ltd). Overnight readings were taken between 0000 h and 0500 h when each participant was asleep; awake readings were taken during a 30-minute window directly after the participant had woken up. Heart rate variability was analyzed with Kubios HRV Premium (version 3.4.1, Kubios) by 2 researchers who were blinded to genotype.

### Glucose Homeostasis and Endocrine Function

Blood samples were obtained in the fasting state and analyzed for lipids, thyrotropin (TSH), free thyroxine (FT4), free 3,5,3′-triiodothyronine, luteinizing hormone, follicle-stimulating hormone, estradiol, testosterone, and cortisol with the use of standard assays. In addition, an oral glucose tolerance test examining the glucose and insulin response to 75-g oral glucose load over 180 minutes was performed in 15 adult variant carriers and 14 controls who did not have a diagnosis of type 2 diabetes.

### Adipose Tissue Biopsies

In 13 case participants and 14 age-, sex-, and BMI-matched controls who consented to the procedure, subcutaneous adipose tissue (SAT) biopsies were obtained from the left iliac fossa, using a nondiathermy surgical biopsy method, under local anesthesia (1% lidocaine with 1:200 000 adrenaline). A 2- to 3-cm skin incision was made, skin flaps were raised on either side of the incision, and the superficial fascia incised. Approximately 2 g of adipose tissue was excised and washed in phosphate-buffered saline. Separate sections (4 μm) of SAT were stained with hematoxylin and eosin (H&E) and Picosirius red and scanned using the Axio Scan.Z1 slide scanner (Zeiss) into whole digital images. To evaluate adipocyte morphology and measure adipocyte surface area, H&E-stained slides were processed using HALO image analysis software (Indica Labs). All adipocytes in the section were included in the analysis; adipocyte surface area was measured using the inbuilt Vacuole v2.2 algorithm. Scoring of anonymized samples from cases with *SRC-1* variants and controls (UK and Paris groups) was performed by the same observer (P.B.L.). Values were used to generate a frequency distribution with a bin width of 500 μm. To quantify adipose tissue fibrosis, Picosirius red–stained slides were assessed using the fibrosis score of adipose tissue (FAT score) (18) by an experienced observer (P.B.L.) blinded to the identity of the samples. Severe adipose tissue fibrosis was defined as a FAT score greater than or equal to 2 as previously described (18).

### De Novo Lipogenesis

We measured DNL in 14 case participants and 16 age-, sex-, and BMI-matched controls. Fasting blood samples were collected at 2000 h, and a priming dose of deuterium-labeled water (3 ml × 0.5 × body weight [kg] for females; 3 mL × 0.6 × body weight [kg] males) was made up to 200 mL with tap water and provided in 2 equal portions at 2000 h and 2200 h. Ad libitum deuterated water (4.5 ml/L) was provided overnight; no food was permitted. A blood sample was collected at 0800 h the following morning. Participants were then provided with breakfast comprising 50% of energy requirements (60% carbohydrate, 30% fat, 10% protein), a further portion of deuterated water at 1000 h and a final blood sample collected at 1200 h.

Total plasma lipids were extracted using chloroform-methanol and triglycerides separated by solid-phase extraction (19). Fatty acid methyl esters were prepared using methanolic sulfuric acid and fatty acid relative abundance (mol %) was determined by gas chromatography (19). Fasting and postprandial hepatic DNL was assessed based on the incorporation of deuterium from ^2^H_2_O in plasma water (Finnigan GasBench-II, Thermo Fisher Scientific) into plasma TAG palmitate using gas chromatography–mass spectrometry, monitoring ions with mass-to-charge ratios of 270 (M + 0) and 271 (M + 1) (20). Background isotopic enrichment in plasma water was measured in a fasting blood sample taken before participants consumed deuterated water. Plasma metabolites (3-hydroxybutyrate, triglyceride, nonesterified fatty acids [NEFAs]) were analyzed enzymatically (ILab 650 clinical chemistry; Werfen).

### Statistical Analysis

Statistical analysis was performed using R and GraphPad prism v9 statistical packages. All data sets were checked for normality of distribution using the Shapiro-Wilk, Kolmogorov-Smirnov tests, and equality of variance using the *F* test. Data sets with normal distributions and equal variance were compared using an unpaired *t* test, and nonparametric data were compared using the Mann-Whitney *U* test. Where multiple comparisons were performed, the Holm-Sidak correction for multiple comparisons was used to compute the adjusted *P* value. Statistical tests are 2-tailed unless otherwise stated and statistical significance of an individual test was declared at *P* less than .05.

## Results

### Identification of Rare Variants in *Steroid Receptor Coactivator-1*

In this study, we performed exome sequencing and targeted resequencing of 2462 people with severe obesity and identified 23 rare heterozygous variants in *SRC-1* (Fig. 1B); a number of variants were found in multiple unrelated individuals and some were present in publicly available exomes (Table 1). In a previous study, we identified 15 variants in 16 unrelated individuals among 2548 people with severe obesity studied using the same methods (6) and showed they caused an LOF in cells using a POMC reporter assay (see Table 1). While we did not perform functional studies of the 23 variants identified in this new study, the variants identified here affect the domain known to interact with STAT3 and the domain that interacts with NHRs (nuclear receptor interacting domain, NRID) (see Fig. 1B). We have previously shown that variants affecting these domains can cause an LOF (6).

**Table 1.** Steroid receptor coactivator-1 (*SRC-1*) variants identified in a cohort with severe obesity (Genetics of Obesity Study)

SRC-1 variant	Nucleotide change	rs No.	No. of probands with variant	gnoMAD allele frequencies							
				European (non-Finnish)	European (Finnish)	Latino/admixed American	East Asian	South Asian	African/African American	Ashkenazi Jewish	Other
E210K	c.628G>A	rs573124083	1	3.517 × 10^–5^	–	–	–	–	6.152 × 10^–5^	–	–
T377N	c.1130C>A	rs139389349	7	1.787 × 10^–4^	–	–	–	–	4.007 × 10^–5^	–	1.387 × 10^–4^
N378K	c.1134T>A	rs1015634733	1	4.408 × 10^–5^	–	–	–	–	–	–	–
M381R	c.1142T>G	–	1	–	–	–	–	–	–	–	–
R385Q	c.1154G>A	rs776205465	1	7.751 × 10^–6^	–	3.106 × 10^–4^	–	3.267 × 10^–5^	–	–	–
S389L	c.1166C>T	rs764983598	1	6.974 × 10^–5^	–	2.822 × 10^–5^	5.012 × 10^–5^	–	–	–	–
H431R	c.1292A>G	rs202008308	5	4.725 × 10^–4^	–	–	–	–	8.012 × 10^–5^	–	–
G439R	c.1315G>A	rs371609618	1	8.796 × 10^–6^	–	–	–	–	6.152 × 10^–5^	–	–
Q448K	c.1342C>A	rs762401650	1	2.638 × 10^–5^	–	–	–	–	–	–	–
Q463H	c.1389G>T	–	1	–	–	–	–	–	–	–	–
L541S	c.1622T>C	rs148155916	1	8.812 × 10^–5^	–	–	–	–	–	–	–
N549S	c.1646A>G	rs541293975	3	3.102 × 10^–5^	–	–	–	5.751 × 10^–3^	4.096 × 10^–5^	–	4.183 × 10^–4^
S557T	c.1670G>C	rs772308327	1	5.428 × 10^–5^	–	–	–	–	–	–	–
S565N	c.1694G>A	rs889784030	1	2.327 × 10^–5^	–	–	–	3.266 × 10^–5^	–	–	–
R572S	c.1716A>T	rs142018995	6	1.513 × 10^–3^	1.990 × 10^–4^	2.089 × 10^–3^	–	–	3.249 × 10^–4^	–	3.482 × 10^–3^
Q597P	c.1790A>C	rs1459123790	1	1.556 × 10^–5^	–	–	–	–	–	–	–
S603C	c.1807A>T	–	1	–	–	–	–	–	–	–	–
D710H	c.2128G>C	rs149214507	1	9.316 × 10^–5^	–	5.647 × 10^–5^	–	–	–	–	1.389 × 10^–4^
A715T	c.2143G>A	rs752190418	1	8.816 × 10^–6^	–	–	–	–	–	–	–
S738L	c.2213C>T	rs1486480744	1	1.762 × 10^–5^	–	–	–	–	–	–	–
M791V	c.2371A>G	rs1034065067	1	–	–	5.855 × 10^–5^	–	–	–	–	–
Q804R	c.2411A>G	rs758160275	1	–	–	1.844 × 10^–5^	–	–	–	–	1.899 × 10^–4^
A857T	c.2569G>A	rs145705009	4	7.685 × 10^–4^	–	–	–	6.546 × 10^–4^	8.013 × 10^–5^	–	5.551 × 10^–4^
T979P	c.2935A>C	–	1	–	–	–	–	–	–	–	–
M984T	c.2951T>C	rs151084207	1	8.795 × 10^–6^	–	–	–	–	6.152 × 10^–5^	–	–
P988S	c.2962C>T	rs763384268	1	8.794 × 10^–6^	–	–	–	–	–	–	–
P1034L	c.3101C>T	-	1	–	–	–	–	–	–	–	–
T1083A	c.3247A>G	rs1271468598	1	–	–	–	–	–	–	–	–
N1142K	c.3426C>A	–	1	–	–	–	–	–	–	–	–
N1212K	c.3636C>G	rs1306789226	2	8.804 × 10^–6^	–	–	–	–	–	–	–
Q1231E	c.3691C>G	–	1	–	–	–	–	–	–	–	–
V1238I	c.3712G>A	rs56099330	1	7.872 × 10^–6^	–	8.930 × 10^–5^	–	–	2.211 × 10^–3^	–	1.418 × 10^–4^
S1250I	c.3749G>T	rs754718198	1	8.798 × 10^–6^	–	–	–	3.267 × 10^–5^	–	–	–
P1257L	c.3770C>T	rs1219997834	1	8.794 × 10^–6^	–	2.892 × 10^-5^	–	3.267 × 10^–5^	–	–	–
Y1277C	c.3830A>G	rs751254362	1	3.519 × 10^–5^	–	–	–	–	–	–	–
T1326M	c.3977C>T	rs759588390	1	3.244 × 10^–4^	–	–	–	–	–	–	–
N1332S	c.3995A>G	rs150066931	6	9.757 × 10^-4^	1.194 × 10^-4^	5.361 × 10^-4^	–	2401 × 10^-2^	4.008 × 10^-5^	9.066 × 10^-3^	4.016 × 10^-3^
L1376P	c.4127T>C	rs201252444	1	5.42 × 10^-5^	–	1.185 × 10^-3^	–	–	–	9.643 × 10^-5^	1.385 × 10^-3^

Variant numbering based on Ensembl transcript ID ENST00000406961. Allele frequencies obtained from gnoMAD v2.1 (https://gnomad.broadinstitute.org/).

**Figure 2.**
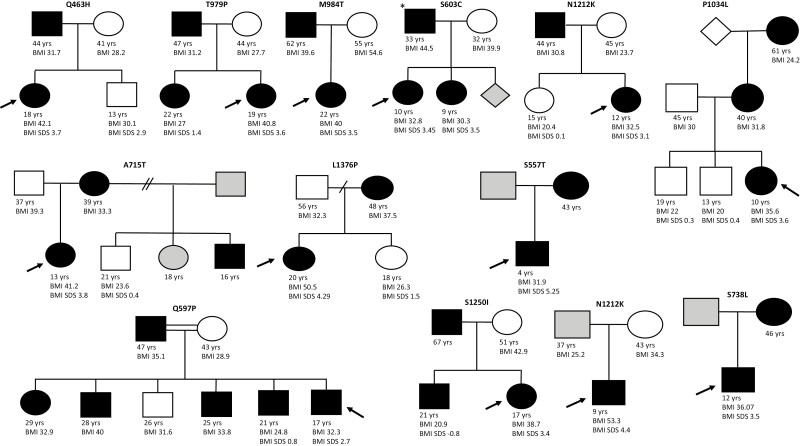
Pedigrees of *SRC-1* cases. Cosegregation of *SRC-1* variants is shown where male (squares) and female (circles) family members consented to genotyping. Heterozygous (filled) and wild-type (empty) *SRC-1* variant carriers indicated; some individuals were not available for genotyping (gray). Body mass index (BMI) (> 25 kg/m2 = overweight; > 30 kg/m2 = obesity) for adults and BMI SDS for children are shown where available. Arrow indicates the proband. Rhombus = unknown. * = Homozygous. SRC-1, steroid receptor coactivator-1.

We performed family cosegregation studies and found that rare variants in *SRC-1* did not consistently cosegregate with severe obesity in a mendelian manner (Fig. 2). This was true even for variants shown to cause a clear LOF in cells previously (6). We conclude that rare *SRC-1* variants are not fully penetrant but rather may interact with other genetic and/or environmental factors to modulate the phenotype. These findings align with those reported for other obesity-associated genes (*MRAP2*, *KSR2*, and *PHIP*) (10, 21, 22), where rare heterozygous variants are associated with obesity but are not always causative, in contrast to the classical monogenic obesity syndromes that follow an autosomal recessive (*LEP*, *LEPR*, *POMC*, and *PCSK1*) or dominant (*MC4R*, *SIM1*, and *GNAS*) mode of inheritance (1).

### Clinical Features Seen in a Cohort of Patients With Steroid Receptor Coactivator-1 Deficiency

**Figure 3.**
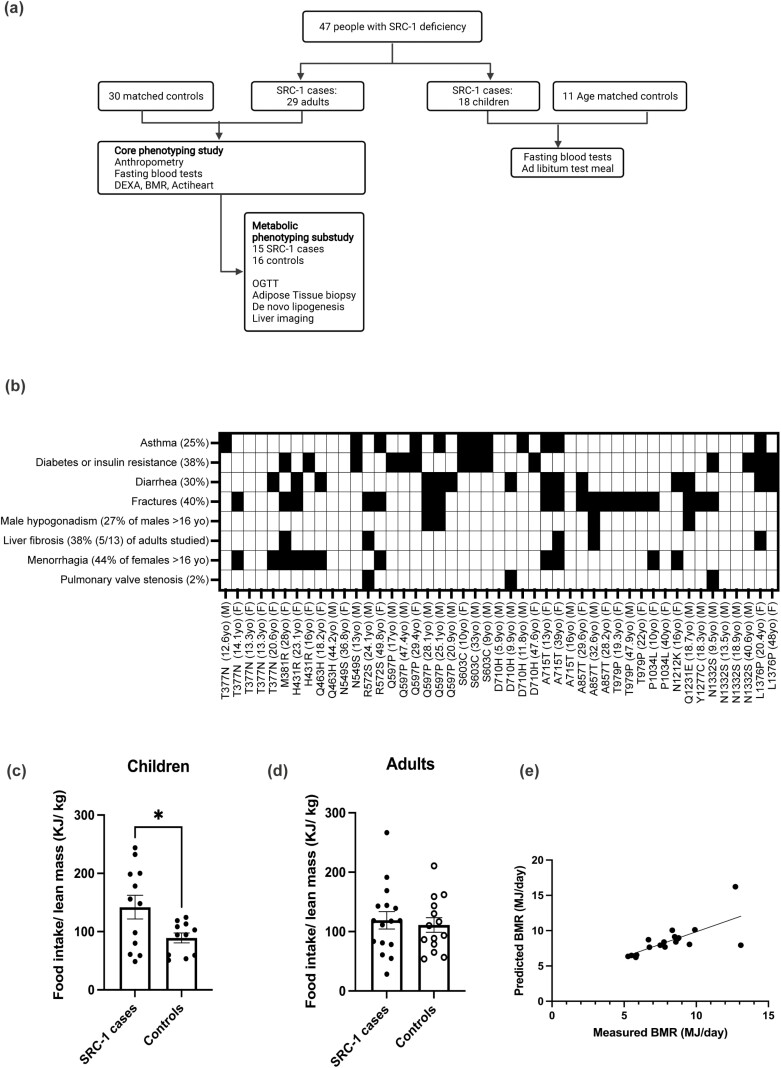
Clinical features of *SRC-1* variant carriers. A, Forty-seven people with *SRC-1* variants consented to phenotypic studies. Twenty-nine adult *SRC-1* cases and 30 age-, sex-, and body mass index–matched controls underwent core phenotyping studies; a subset of 15 *SRC-1* cases and 16 controls consented to additional metabolic phenotyping studies. Eighteen children with variants in *SRC-1* were compared to 11 age-matched controls. B, Additional clinical features seen in *SRC-1* variant carriers; details of age, sex and *SRC-1* variant are shown. C and D, Ad libitum energy intake assessed using a 18-MJ meal of known macronutrient content after an overnight fast in C, children, and D, adults; food intake expressed per kilogram lean body mass measured by dual-energy x-ray absorptiometry. E, Basal metabolic rate (BMR; megajoules, MJ/d) determined by indirect calorimetry after an overnight fast. Measured BMR was compared to BMR predicted by age- and sex-specific equations. SRC-1, steroid receptor coactivator-1.

We invited all probands with rare variants in *SRC-1* and their affected family members to take part in clinical studies. Twenty-nine adults and 18 children (age < 18 years) with rare variants in *SRC-1* consented to take part (Fig. 3A). Twenty-nine adult *SRC-1* variant carriers were studied using a core phenotyping protocol (Table 2); mean (± SD) age 31.4 ± 2.1 years (range, 18.2-49.8 years); their data were compared to 30 controls (mean (± SD) age 30.9 ± 1.7 years (range, 19.0-48.4 years, *P* = .94). The mean BMI of the adult *SRC-1* variant carriers was 41.8 ± 13.3 (23.3-74.9) and controls 43.2 ± 12.7 (26.8-85.2, *P* = .62). A subset of these individuals, 15 SRC-1 case participants and 16 controls, consented to take part in a metabolic substudy that involved liver imaging, adipose tissue biopsies, and stable-isotope studies of DNL (Fig. 3A and Table 2).

**Table 2.** Steroid receptor coactivator-1 (*SRC-1*) variant carriers included in clinical studies

Study	*SRC-1* variant	Age, y	Sex	BMI	BMI SDS
Core phenotyping	Q463H^*a*^	18.2	F	42.1	
Core phenotyping	Q463H	44.2	M	31.7	
Core phenotyping	N549S	36.8	F	28.7	
Core phenotyping	Q597P	47.4	M	35.1	
Core phenotyping	Q597P	29.4	F	32.9	
Core phenotyping	Q597P	28.1	M	40.0	
Core phenotyping	Q597P	25.1	M	33.8	
Core phenotyping	Q597P	20.9	M	24.8	
Core phenotyping	S603C	33.0	M	44.5	
Core phenotyping	A857T	28.2	F	35.5	
Core phenotyping	T979P^*a*^	19.3	F	40.8	
Core phenotyping	T979P	47.9	M	31.2	
Core phenotyping	T979P	22.0	F	27.0	
Core phenotyping	L1376P	48.0	F	37.5	
Core and metabolic	T377N^*a*^	20.6	F	50.6	
Core and metabolic	M381R^*a*^	28.0	F	53.1	
Core and metabolic	H431R^*a*^	23.1	F	50.9	
Core and metabolic	R572S^*a*^	24.1	M	52.7	
Core and metabolic	R572S	49.8	F	48.3	
Core and metabolic	D710H	47.6	F	50.6	
Core and metabolic	A715T	39.0	F	33.3	
Core and metabolic	A857T^*a*^	29.6	F	57.5	
Core and metabolic	A857T^*a*^	32.6	M	74.8	
Core and metabolic	P1034L	40.0	F	31.8	
Core and metabolic	Q1231E^*a*^	18.7	M	55.6	
Core and metabolic	Y1277C^*a*^	18.3	M	38.5	
Core and metabolic	N1332S	18.9	M	23.3	
Core and metabolic	N1332S	40.6	M	29.7	
Core and metabolic	L1376P^*a*^	20.4	F	50.5	
Pediatric	T377N	12.6	M	29.3	2.1
Pediatric	T377N^*a*^	14.1	F	46.3	4.1
Pediatric	T377N^*a*^	13.3	F	41.1	2.8
Pediatric	T377N	13.3	F	32.9	2.4
Pediatric	H431R^*a*^	16.0	F	52.3	4.4
Pediatric	N549S^*a*^	13.0	M	35.1	3.3
Pediatric	Q597P^*a*^	17.0	M	32.3	2.7
Pediatric	S603C^*a*^	10.0	F	32.8	3.5
Pediatric	S603C	9.0	M	30.3	3.5
Pediatric	D710H	5.9	M	16.8	1.0
Pediatric	D710H^*a*^	9.9	M	27.9	2.2
Pediatric	D710H	11.8	M	18.5	0.2
Pediatric	A715T^*a*^	13.0	F	41.2	3.8
Pediatric	A715T	16.0	M	–	–
Pediatric	P1034L^*a*^	10.0	F	35.6	3.6
Pediatric	N1212K^*a*^	16.0	F	36.8	2.2
Pediatric	N1332S^*a*^	9.5	M	33.6	2.7
Pediatric	N1332S	13.5	M	25.9	1.7

Forty-seven *SRC-1* variant carriers (22 probands, 25 family members) consented to participate in physiological studies. All 29 adults participated in a core phenotyping protocol; a subset of 15 adults participated in metabolic studies. Eighteen children were studied on a limited pediatric protocol.

Abbreviations: –, information not available; BMI, body mass index; F, female, M, male.

^*a*^Proband.

A high proportion (40%; n = 19) of *SRC-1* variant carriers had a history of fractures in response to minor injuries in contrast to patients with other genetic obesity syndromes (1) (Fig. 3B). Whole-body BMD was comparable in cases and controls (BMD *Z* score 0.6 ± 0.2 vs 0.7 ± 0.2, respectively). While none of the *SRC-1* variant carriers had thyroid function tests suggestive of RTH (high TSH/high FT4, Table 3), interestingly, T4 treatment for autoimmune hypothyroidism of the women carrying the L1376P and A715T variants was complicated by failure to adequately suppress TSH, despite high-dose (> 200 μg) levothyroxine, unless FT4 levels were above 25 pmol/L (normal range, 10-19.8 pmol/L). We carefully reviewed compliance with T4 therapy. Notably, there were no features of malabsorption or bowel disease at colonoscopy in the patient carrying L1376P *SRC-1*.

**Table 3.** Metabolic and endocrine measurements in adult steroid receptor coactivator-1 (*SRC-1*) variant carriers compared to age-, sex-, and body mass index–matched controls

	Reference range	*SRC-1* cases	Controls
		n = 29	n = 30
% Male		45	50
**Endocrine**			
TSH, mU/L	0.35-5.5	1.9 ± 0.2	2.2 ± 0.2
Free T4, pmol/L	10.0-19.8	14.5 ± 0.3	14.4 ± 0.3
Free T3, pmol/L	3.5-6.5	5.1 ± 0.1	5.1 ± 0.1
**OGTT**			
Fasting glucose, mmol/L	3.5-5.5	4.6 ± 0.1	4.8 ± 0.1
Fasting insulin, pmol/L	0-60	123.8 ± 16.1	110.0 ± 8.4
AUC glucose, mmol min/L		1126 ± 70	1203 ± 96
AUC insulin, pmol min/L		2062 ± 414	1535 ± 314
**Lipids and liver function tests**			
Total cholesterol, mmol/L	0.0-5.2	4.7 ± 0.2	4.4 ± 0.1
Calculated LDL cholesterol, mmol/L	0.0-2.6	2.9 ± 0.2	2.8 ± 0.1
HDL cholesterol, mmol/L	1.0-1.5	1.2 ± 0.06	1.0 ± 0.04
Triglycerides, mmol/L	0.0-1.68	1.5 ± 0.1	1.5 ± 0.1
Albumin, g/L	35-52	37.2 ± 0.5	36.2 ± 0.6
ALT, U/L	10-49	29.3 ± 2.4	34.7 ± 2.7
ALP, U/L	53-129	77.7 ± 3.7	77.2 ± 4.9
Bilirubin, μmol/L	0-20	10.4 ± 1.1	11.4 ± 1.3
**DNL ~**			
Fasting DNL, %		6.8 ± 0.9	7.0 ± 0.7
Fasting DNL, μmol/L		104.8 ± 20.9	89.2 ± 14.9
Postprandial DNL, %		12.3 ± 0.8	13.4 ± 1.5
Postprandial DNL, μmol/L		292.7 ± 45.8	283.2 ± 54.9
Fasting 3-hydroxybutyrate, μmol/L		67.5 ± 12.9	50.4 ± 10.0
Postprandial 3-hydroxybutyrate, μmol/L		27.9 ± 8.5	15.1 ± 2.1
Fasting NEFAs, μmol/L		517.4 ± 39.6	512.2 ± 36.8
Postprandial NEFAs, μmol/L		191.7 ± 15.6^*a*^	137.1 ± 18.7
**HR variability and BP**			
Mean HR awake, bpm		66 ± 1.8	68 ± 1.9
RMSSD awake, ms		61.3 ± 9.0	50.5 ± 7.4
HF awake, ms²		2010 ± 803	1521 ± 464
LF awake, ms²		2800 ± 1292	1109 ± 179
LH/HF ratio awake		2.5 ± 0.8	2.5 ± 0.5
Mean HR overnight, bpm		68 ± 2.1	68 ± 1.8
RMSSD overnight, ms		59.5 ± 7.2	54.9 ± 7.2
HF overnight, ms²		1799 ± 441	1369 ± 388
LF overnight, ms²		1502 ± 400	1638 ± 318
LH/HF ratio overnight		1.5 ± 0.3	3.0 ± 0.7
SBP, mm Hg	90-140	125 ± 3	127 ± 3
DBP, mm Hg	60-90	75.9 ± 2.2	75.7 ± 2.0

Values are presented as mean ± SEM.

Abbreviations: ~ a subset of people underwent detailed metabolic phenotyping (see Table 2); ALP, alkaline phosphatase; ALT, alanine aminotransferase; AUC, area under the curve; BP, blood pressure; DBP, diastolic blood pressure; DNL, de novo lipogenesis; HDL, high-density lipoprotein; HF, high frequency; HR, heart rate; LDL, low-density lipoprotein; LF, low frequency; NEFA, nonesterified fatty acid; OGTT, oral glucose tolerance test; RMSSD, root mean square of successive differences; SBP, systolic blood pressure; TSH, thyrotropin.

^*a*^ *P* less than .05; otherwise there was no statistically significant difference between cases and controls.

Menstrual irregularities are common in women with obesity. We found that 13% (2/15) of women (age 16-50 years) with *SRC-1* variants had a history of polycystic ovary syndrome (with hyperandrogenism and hirsutism); this was comparable to severely obese women with MC4R deficiency (9%; 2/22) of comparable BMI. In addition, 8 out of 18 female *SRC-1* variant carriers older than 16 years (44%) reported menorrhagia with persistent bleeding (see Fig. 3B). One female *SRC-1* variant carrier was diagnosed with complex atypical endometrial hyperplasia in her early 20s that responded well to progestogen therapy. One female variant carrier of the L1376P variant had primary amenorrhea. Women with *SRC-1* variants were more likely to be prescribed tranexamic acid for persistent bleeding (13.5%; 4/15) than women with MC4R deficiency (0 of 22) we have studied.

Four out of 12 adult male *SRC-1* variant carriers had low testosterone and gonadotropin levels consistent with hypogonadotropic hypogonadism (see Fig. 3B). Three out of these 4 variant carriers had signs of primary hypogonadism (undescended testis, reduced penile length). In comparison, 3 out of 8 male controls had low testosterone and gonadotropin levels, consistent with secondary hypogonadotropic hypogonadism. None of the variant carriers had overt clinical signs or symptoms of mineralocorticoid or corticosteroid hormone resistance.

A number of *SRC-1* variant carriers had been diagnosed with hepatic fibrosis or cirrhosis at a young age (see Fig. 3B). Liver function tests were within the normal range in case and control participants (see Table 3); liver volume determined by T2-weighted MRI (2126 ± 545 cm^3^ in case participants v 2388 ± 661 cm^3^ in controls, *P* = .29) and liver fat assessed using MRI-PDFF was comparable in case and control individuals (7.1% vs 10.9%, respectively, *P* = .38). We found that 5 out of 13 (38%) adult *SRC-1* variant carriers had significant hepatic fibrosis compared to 2 out of 13 (15%) adult controls; odds ratio equal to 3.4 (*P* = .38). Fourteen *SRC-1* variant carriers (30%) reported frequent and/or persistent diarrhea, described as pale, loose stools, typically after fatty food. There was no biochemical evidence of macronutrient or micronutrient malabsorption (see Table 3). These features could be consistent with bile acid malabsorption; further studies will be required to test this.

Twelve *SRC-1* variant carriers (25%) had moderate to severe asthma requiring home nebulizers, frequent courses of oral corticosteroids, and/or hospital admissions (see Fig. 3B). STAT3 is a major regulator of the differentiation and function of Th17 cells, a subset of CD4^+^ T cells implicated in inflammation in patients with severe asthma. Three variant carriers had pulmonary valve stenosis, a rare congenital abnormality (population prevalence 3/10 000 births). Further studies will be needed to explore the mechanisms underlying these observations.

### Body Fat Distribution, Energy Intake, and Expenditure

Children demonstrated increased food intake at an 18-MJ ad libitum test meal compared to age-matched controls (Fig. 3C); food intake was not increased in adults with *SRC-1* variants compared to controls (Fig. 3D). In contrast to findings in SRC-1–deficient mice, there was no deficit in measured basal metabolic rate, which correlated tightly with that predicted by age, sex and body composition (*R* = 0.6989, *P* = .0013, Fig. 3E). Heart rate and heart rate variability (markers of autonomic nervous system tone) were comparable between the groups (see Table 3). We quantified the ratio of visceral adipose tissue (VAT) to SAT using MRI. VAT:SAT was comparable in *SRC-1* variant carriers and controls (1.49 ± 0.91 in cases vs 1.27 ± 0.48 in controls, *P* = .9).

### Glucose and Lipid Homeostasis

**Figure 4.**
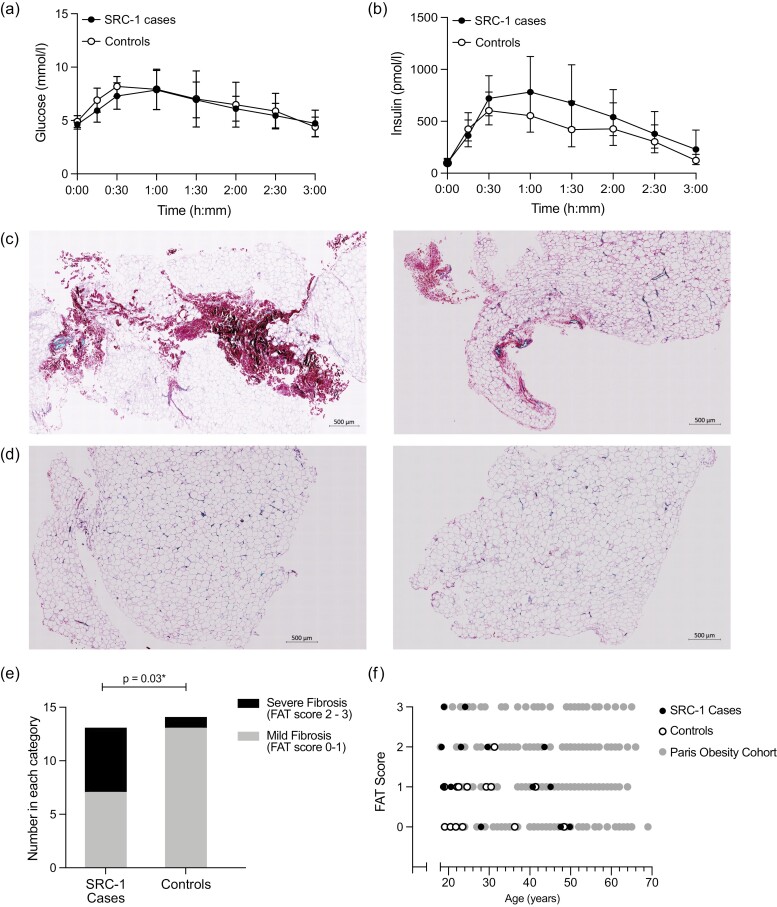
Glucose homeostasis and adipose tissue morphology in *SRC-1* variant carriers. A and B, Insulin and glucose levels during a 75-g oral glucose tolerance test over 180 minutes in SRC-1 cases and controls. C and D, Representative histology of subcutaneous adipose tissue biopsies taken from *C, SRC-1* variant, carriers, and D, controls, stained for collagen, consistent with fibrosis score of adipose tissue (FAT) 3 and 0, respectively. Picosirius red staining, scale bar = 500 μm. E, *SRC-1* variant carriers (cases) have a statistically significantly higher proportion of severe adipose tissue fibrosis (FAT 2-3), compared to age-, sex-, and body mass index–matched controls. F, Distribution of FAT score by age in *SRC-1* variant carriers (black circles), matched controls (open circles), and a large cohort of individuals with severe obesity undergoing bariatric surgery (gray circles, “Paris obesity cohort”). SRC-1, steroid receptor coactivator-1.

Two out of 29 *SRC-1* variant carriers had a diagnosis of type 2 diabetes. Although SRC-1 has been implicated in hepatic glucose production (23), we did not find any differences in fasting glucose or insulin levels or in the area under the curve during a 75-g oral glucose tolerance test between *SRC-1* variant carriers and controls (Fig. 4A and 4B; see Table 3). Fasting lipid profiles were within the normal range in case and control participants (see Table 3). We measured fasting and postprandial DNL using deuterated water. There was no difference between cases and controls in fasting hepatic DNL, expressed as a percentage or a concentration (see Table 3). With meal consumption, DNL increased in both groups to a similar extent (see Table 3). We found no difference in fasting plasma concentrations of 3-hydroxybutyrate, a marker of hepatic fatty acid oxidation, between case and control participants (see Table 3). As expected, there was a statistically significant decrease in plasma 3-hydroxybutyrate concentrations postprandially in both groups (see Table 3). Fasting NEFA concentrations were not statistically significantly different between case and control individuals (see Table 3). While postprandial NEFA concentrations decreased in both groups, this decline was attenuated in case vs control participants (191.7 ± 15.6 µmol/l vs 137.1 ± 18.7 µmol/L, respectively, *P* = .02), which may suggest impaired insulin sensitivity of the adipose tissue.

### Adipose Tissue Morphology and Fibrosis

Omental and SAT can both undergo fibrosis in people with obesity (24, 25). Increased adipose tissue fibrosis is associated with inflammation (26) and may lead to decreased tissue plasticity and thus a failure to expand fat mass with sustained weight gain (27, 28). In addition, adipose tissue fibrosis is associated with reduced weight loss following bariatric surgery in people with severe obesity (18, 29). *SRC-1* variant carriers had a higher proportion of smaller adipocytes (500 µm^2^) compared to controls (21.7 ± 4.3% in case individuals vs 19.6 ± 6.0% in control individuals, adjusted *P* = .02). Further analysis of the tissue demonstrated marked fibrosis in biopsies taken from several case participants (Fig. 4C and 4D). The degree of fibrosis was assessed using a previously reported FAT score (18). A greater proportion of *SRC-1* variant carriers had severe fibrosis (FAT score 2-3) compared to age-, sex-, and BMI-matched controls (46.2% vs 7.1%, *P* = .03) (Fig. 4E). There was no correlation between FAT score and BMI in either group. In addition, we compared our findings to those from a cohort of 265 similarly obese patients (mean BMI ± SD 46.3 ± 14.1 vs 46.6 ± 6.4, *P* = .93) attending for bariatric surgery in Paris, France (data on 183 of these patients were previously published) (18). The prevalence of severe fibrosis was comparable in both groups (46.2% v 39.2%, *P* = .7), although the Paris control group was statistically significantly older than the *SRC-1* variant carriers in our study (31.4 ± 12.1 vs 42.9 ± 12.1 years, respectively, *P* < .01) (Fig. 4F).

## Discussion

In this study, we describe the clinical features seen in people with severe obesity carrying rare variants in *SRC-1*. In keeping with findings in patients with other genetic disorders affecting the leptin-melanocortin pathway, *SRC-1* variant carriers experience hyperphagia in childhood, but have a normal basal metabolic rate and mild insulin resistance. These findings are consistent with previous findings showing that SRC-1 interacts with phosphorylated STAT3 to modulate leptin-mediated POMC transcription and with findings in a mouse model of a human LOF *SRC-1* variant (6).

We observed a very high prevalence of multiple fractures with minimal trauma in males and females starting from childhood, in contrast to findings in patients with other genetic obesity syndromes such as MC4R deficiency (30). SRC-1 plays an important role in the development and maintenance of bone density, primarily through its interaction with the estrogen receptor (31, 32). The skeletal effect of *SRC-1* variants may also be mediated by resistance to the vitamin D receptor and the TH receptor, which function as heterodimers with retinoid X receptor. Although we did not observe a reduction in whole-body BMD in SRC-1 case vs control participants, it is important to note that deletion of *SRC-1* results in trabecular osteopenia in male and female mice (13). Trabecular bone accounts for only 20% of total bone mass in humans. Targeted spine, femur, femoral neck, and distal radius DEXA scans and/or high-resolution peripheral quantitative computed tomography or bone histology may be needed to investigate the effect of SRC-1 deficiency on bone architecture.

SRC-1 enhances TH receptor–mediated signaling (33). *SRC-1* knockout mice display RTH with a 2.5-fold elevation of TSH levels, despite a 50% increase in free TH levels (11). Whilst none of the *SRC-1* variant carriers in our study had overt classical RTH, treatment of concomitant autoimmune hypothyroidism was complicated by failure to suppress TSH in 2 variant carriers, despite high-dose T4. There was no suggestion of noncompliance or intermittent compliance, and there were no features of malabsorptive disorders such as celiac disease or bowel disease. Interestingly, the decline of TSH observed in mice treated with L-triiodothyronine is blunted in *SRC-1* knockout mice, indicating that SRC-1 enhances the sensitivity by which TH downregulates TSH (11, 34). Studies in larger cohorts of patients will be needed to establish whether partial RTH, as seen in *SRC-1* knockout mice, is a feature in some people with *SRC-1* variants.

Multiple female *SRC-1* variant carriers reported severe menorrhagia that significantly affected their quality of life. SRC-1 is expressed in the endometrium; its expression increases during menstruation both in the glandular epithelium and stroma, where it may play a role in endometrial remodeling (35, 36) that depends on the balance of estrogen and progesterone signaling (12, 37). Interestingly, a truncated SRC-1 isoform has been implicated in the pathophysiology of endometriosis (38). The complex atypical endometrial hyperplasia of one of the *SRC-1* variant carriers did respond to progestogens, suggesting that any subtle defects in progesterone signaling due to *SRC-1* variants may be overcome by pharmacotherapy with progesterone.

Generally, increased adipose tissue mass is characterized by adipocyte hypertrophy followed by hyperplasia. In chronic obesity (as seen in the older patients studied in the Paris cohort), adipose tissue undergoes major remodeling, resulting in inflammation and fibrosis, which represents a physical constraint to adipose tissue expansion (39). Studies in rodents (40) (and insights from patients with lipodystrophy) (41) indicate that this reduction in adipose expandability promotes the deposition of excess lipid in liver and skeletal muscle leading to metabolic complications (42). Here, glucose tolerance was comparable in case and control individuals tightly matched for age and BMI. Whether increased adipose tissue fibrosis in young patients with *SRC-1* variants predisposes them to the early development of metabolic complications such as type 2 diabetes and fatty liver disease is an important question that needs to be addressed in larger longitudinal studies. The observation that several patients have hepatic fibrosis (but not steatosis) is not readily explained and will require further investigation and long-term monitoring.

Larger studies will be needed to test for genotype-phenotype correlations. While a common variant in the gene encoding SRC-1 (*NCOA1*) has been associated with reduced hip and lumbar BMD in women receiving tamoxifen treatment (32), to date variants at this locus have not been associated with BMI in genome-wide association studies.

In conclusion, the clinical spectrum associated with *SRC-1* variants is characterized by hyperphagia in childhood and severe obesity and encompasses a range of other metabolic and endocrine features. The interpretation of these findings is challenging because several factors including the functional consequences of different *SRC-1* variants, the presence of residual SRC-1 activity in carriers of heterozygous variants, and compensation by the closely related molecule SRC-2 (7) will contribute to phenotypic heterogeneity. Further functional characterization of the *SRC-1* variants reported here will be needed to establish their pathogenicity. All are very rare, affect highly conserved residues, and are located in domains in which we have previously identified LOF variants. Nonetheless, as seen with the first clinical descriptions of functional variants affecting TH receptor-α and -β and estrogen receptor-α, these findings illustrate important aspects of physiology and pathophysiology. Human variants affecting SRC-1 will be useful molecular tools with which to dissect NHR signaling and its regulation by coactivators and corepressors, studies that will provide insights into underlying disease mechanisms and ultimately inform the clinical investigation and management of patients.

## Data Availability

Restrictions apply to the availability of some or all data generated or analyzed during this study to preserve patient confidentiality. The corresponding author will on request detail the restrictions and any conditions under which access to some data may be provided.

## Abbreviations

ARFI: acoustic radiation force impulse
BMD: bone mineral density
BMI: body mass index
DNL: de novo lipogenesis
DEXA: dual x-ray absorptiometry
FAT: fibrosis score of adipose tissue
FT4: free thyroxine
LOF: loss-of-function
MC4R: melanocortin-4 receptor
MRE: magnetic resonance elastography
MRI: magnetic resonance imaging
NEFA: nonesterified fatty acid
NHR: nuclear hormone receptor
PDFF: proton density fat fraction
POMC: proopiomelanocortin
RTH: resistance to thyroid hormone
SAT: subcutaneous adipose tissue
SRC-1: steroid receptor coactivator-1
STAT3: signal transducer and activator of transcription-3
T4: thyroxine
TH: thyroid hormone
TSH: thyrotropin
VAT: visceral adipose tissue
